# Mechanism of Shugan Yidan fang, a Chinese herbal formula, in rat model of premature ejaculation

**DOI:** 10.1186/s12610-023-00200-3

**Published:** 2023-10-03

**Authors:** Qiang Han, Jun Guo, Renyuan Wang, Jiangminzi Li, Fu Wang, Qinghe Gao, Jiwei Zhang, Hetian Wang, Yin Zeng

**Affiliations:** 1 Department of Andrology, Beijing Hospital of Traditional Chinese Medicine, Capital Medical University, 23 Art Gallery Back Street, Dongcheng District, Beijing, China; 2 Department of Andrology, Xiyuan Hospital of China Academy of Traditional Chinese Medicine, Beijing, 100089 China; 3 Department of Endocrinology, Beijing Hospital of Traditional Chinese Medicine, Capital Medical University, Beijing, 100010 China

**Keywords:** Premature ejaculation, Shugan Yidan fang, Dopamine receptor, Rat, Éjaculation précoce, Shugan Yidan, Récepteur de la dopamine, Rat

## Abstract

**Background:**

Premature ejaculation (PE) is one of the most common forms of sexual dysfunction in men, and multimodal therapeutic regimens should be considered to treat the condition. We developed a Chinese medicine herbal medicine, Shugan Yidan fang that had a significant clinical effect on PE patients, extending the time between penetration and ejaculation. However, the mechanism of this formula remains unclear. There is evidence that PE is associated with peripheral neuropathology, and the actions of dopamine (DA) and 5-hydroxytryptamine (5-HT). The aim of this study was to investigate the mechanism of Shugan Yidan fang’s effect on PE through the relationship between sexual behavioristics and the level of neurotransmitters and dopamine receptors (DARs).

**Results:**

We showed that the male PE groups had a significant PE phenotype compared to healthy rats. Treatment with Shugan Yidan fang improved the behavioristics of the PE rats, and reduced the expression of DAR mRNA and protein while improving dopamine transporter levels.

**Conclusions:**

Our study provided evidence for the beneficial effect of Shugan Yidan fang in PE therapy, and proposed a preliminary potential mechanism for the clinical application of the formula.

**Supplementary Information:**

The online version contains supplementary material available at 10.1186/s12610-023-00200-3.

## Résumé

**Contexte:**

L'éjaculation précoce (EP) est l'une des formes les plus courantes de dysfonction sexuelle chez les hommes, et des régimes thérapeutiques multimodaux doivent être envisagés pour traiter la maladie. Nous avons développé une phytothérapie de médecine chinoise, dénommée Shugan Yidan fang, qui a eu un effet clinique significatif sur les patients atteints d'EP, prolongeant le temps entre la pénétration et l'éjaculation. Le mécanisme d’action de cette formule reste cependant flou. Il existe des preuves que l'EP est associée à une neuropathologie périphérique et aux actions de la dopamine (DA) et de la 5-hydroxytryptamine (5-HT). Le but de cette étude était d'étudier le mécanisme de l'effet de Shugan Yidan sur l'EP à travers la relation entre les comportements sexuels et le niveau de neurotransmetteurs et de récepteurs de la dopamine (DAR).

**Résultats:**

Nous avons montré que les groupes PE mâles avaient un phénotype PE significatif par rapport aux rats sains. Le traitement avec Shugan Yidan fang a amélioré les comportements sexuels des rats PE et a réduit l'expression de l'ARNm DAR et des protéines, tout en améliorant les niveaux de de transporteurs de la dopamine.

**Conclusions:**

Notre étude a fourni des preuves de l'effet bénéfique du Shugan Yidan dans la thérapie de la PE, et a proposé un mécanisme potentiel préliminaire pour l'application clinique de la formule.

## Introduction

Premature ejaculation (PE) is the most common male sexual dysfunction [1], with the prevalence of PE 20%-30% based on the statistics in the Guidelines for the diagnosis and treatment of male sexual dysfunction in Europe [2]. Patients with PE have a shorter ejaculation time, which seriously affects their quality of life. The low frequency of sexual life not only affects the relationship between the partners, but also induces anxiety and depression and other mental disorders in patients [3]. However, the pathophysiology of PE is still not clearly understood.

Recent research showed that PE is associated with peripheral neuropathology [4]. Animal studies have shown that 5-hydroxytryptamine(5-HT) and dopamine (DA) play an essential role in the pathology of PE [5, 6]. However, as a first-line treatment for PE [7], the selective serotonin re-uptake 5-HT inhibitor-, dapoxetine, has been reported to have several side-effects, including nausea, diarrhea and headache. DA, an important neurotransmitter, regulates mental activity and somatic movement through the DA receptor (DAR) and transporter (DAT) [8], which play key roles in the mechanism of ejaculation. The DAT participates in the control of both the extracellular and intraneuronal level of DA homeostasis, thereby providing effective control over the activity of dopaminergic transmission. Studies have demonstrated that D2-like receptors of DAR-D2 and D3 participate in DA secretion [9], with these receptors highly expressed on cerebral nuclei and the nucleus paraventricularis that are associated with regulation of ejaculation. Moreover, studies in rats have shown that pre-treatment of sulpiride, a non-selective DAR antagonist, avoids PE induced by several stimulants [10], while 68.8% patients with PE had a recovery after treatment with the sulpiride [11]. Therefore, we consider that D2/D3 receptors and DAT play important roles in ejaculation, and are associated with the treatment of PE.

Recent studies have shown that traditional Chinese medicine has effective functional properties with low side effects in the treatment of PE [12, 13]. In previous clinical observation, we showed that Shugan Yidan fang had a remarkable clinical effect on PE. Studies have demonstrated that the main active ingredient of Shugan Yidan fang, radix bupleuri,regulates emotions and depression [14], while polygala tenuifolia promotes the release of DA and 5-HT [15].

This paper describes a further study in a rat model of PE that investigated the potential mechanism of Shugan Yidan fang in the therapy of PE. The findings of this study on the relationship between DA/DAR/DAT and the formula will be used to support the application of traditional Chinese medicine for treating PE.

## Materials and methods

**Fig. 1:**
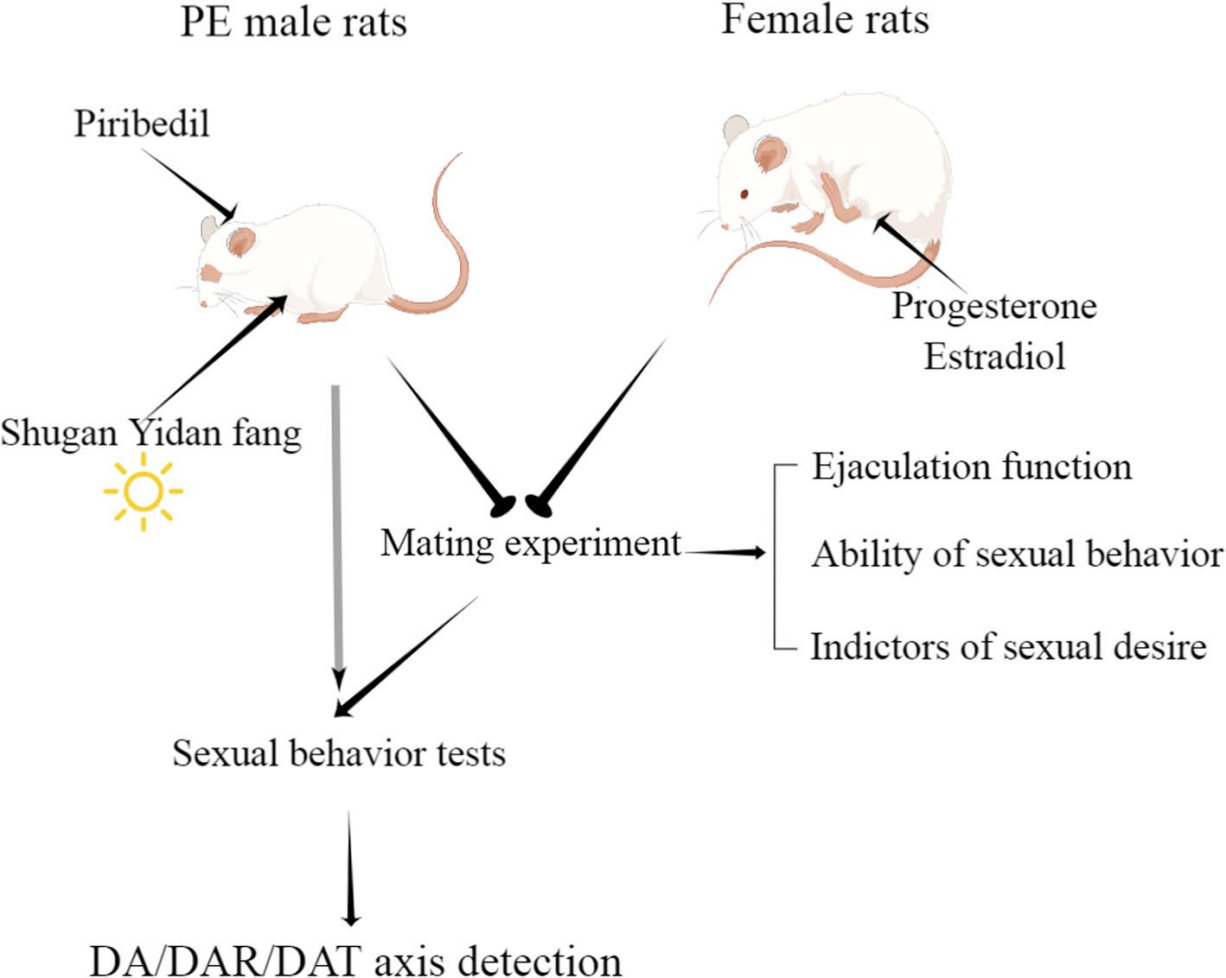
A flowchart for this work. DA, dopamine; DAR, dopamine receptors; DAT, dopamine transporter; PE, premature ejaculation

The model and detection methods used in the study are shown in the flowchart (Fig. 1).

### Animals

Wistar rats weighing 200–250 g, including 60 males and 20 females, were provided by the Experimental Animal Center, Beijing Institute of Traditional Chinese Medicine. The male and female rats were kept in separate cages, with free access to water and food. Animals were raised in a 12-h light/dark cycle environment with a temperature of 18–22℃ and relative humidity of 55%-70%. The study was approved by the ethics committee of the Capital University of Medical, China.

### Premature ejaculation model

Male rats were administered the DAR agonist,—piribedil, once a day at a dose of 5 μg/kg. After 15 days, we started the sexual intercourse test daily for a total of two weeks, that involved screening the PE rat models (3–5 in 30 min).

### Preparation of female rats

Bilateral ovariectomy was performed in rats after they had been anesthetized using pentobarbital sodium, Sodium ampicillin was injected intramuscularly three days after surgery to prevent infection. Two weeks later, estradiol benzoate (2 mg/mL, 10 μl) and progesterone (20 mg/ml, 25 μl) were injected subcutaneously. We screened the female rats for oestrus using a smear prepared from their vaginal discharge.

### Drug administration and grouping

Clinically, Shugan yidan fang including *Bupleurum* 15 g, *Paeony* 15 g, *Codonopsis* 10 g, *Morinda morinda* 15 g, *Pinellia pinellia* 10 g, *Bile south star* 10 g, *Atractyloides* 10 g, *Polygala tenuifolia* 10 g, *Honey-fried licorice root* 6 g, decoction, one dose a day.

A random number table was used to assign the PE rats to receive intragastric administration of ShuganYidan fang at either low (8.75 g/kg/d), medium (17.5 g/kg/d), or high (35 g/kg/d) doses for 4 weeks. The rats were divided into six groups, including normal male rats (control) that received intragastric administration of 10 mL/kg/d distilled water, PE model rats, PE model rats receiving 0.3 mg/kg/d sulpiride (DAR antagonist), and PE model rats receiving either low, medium, or high dose of ShuganYidan fang. The trials were carried out using a double-blinded \ process, that involved the people in charge of rat feeding, treatment, and data analysis not knowing the gouping of the rats.

### Mating experiment

The PE model rats were placed into cages with somber and red color light irradiation. After 5 min the female rats in oestrus were placed in;the cage and the behavior of the rats was observed for 30 min. The following parameters were recorded: Ejaculation function including ejaculation latency (time from first insertion to ejaculation), ejaculation frequency (number of ejaculations of the male rats within 30 min), ability of sexual behavior including intromission latency (time of first insertion by the male rats after placement of the females in the cage), intromission frequency (number of vaginal insertions by male rats into the female rats within 30 min). Indictors of sexual desire including mount latency (time that the male rats first licked the perineum, anus, or mouth of the female rats after their placement in the cage) and mount frequency (the number of times a male climbs on a female within 30 min).

### Hypothalamus separation and detection

Hypothalamus tissues were obtained from the rats after the last mating test (6 rats in each group), and the levels of DA and 5-HT were then tested using reversed phase high-performance liquid chromatography (RP-HPLC). Meantime, Reverse transcription-quantitative real-time PCR (RT-qPCR), Western Blotting (WB) and enzyme-linked immunosorbent assays (ELISA) were used to detect the expression of adrenergic receptor (Alpha 1α), DAR D2/D3 and the DAT.

#### Quantitative real-time PCR

qPCR was performed as described previously. In brief, total RNA was extracted from the hypothalamus tissues using the Trizol method. Total RNA concentration was defined by the OD_260_/OD_280_ value. RNA was reverse transcribed to cDNA by the PrimeScriptTM RT reagent kit (Takara, Japan) according to the manufacturer’s instructions. qPCR was performed using SYBR® Premix Ex Taq™ II (Takara, Japan) in a quantitative fluorescence PCR instrument (96 light cycler, Roche). The cycle programs of the PCR were 95℃ for 30 s, 40 cycles of 95℃ for 5 s and 60℃ for 34 s, followed by 95℃ for 15 s, 60℃ for 60 s and 95℃ for 15 s. The sequences of the primers used were: DAT-F: CCTCTTTGGCGTGCTCATTG, DAT-R: TGCTGACCACGACCACATAC. D2-F: CGGCCTACATAGCAACCCTG, D2-R: TTTCTGCGGCTCATCGTCTT. D3-F: CACTCGACAGAACAGCCAGT, D3-R: GGCTGCAGGTGTGACAAAAG. Actin-F: GGCTGTGCTATCCCTGTACG, actin-R: AGGTAGTCAGTCAGGTCCCG. Adra1α-F: GGAGTCAGCAGTGCCAAGAATAAGA, Adra1α-R: AGCCAGCAGAGGACGAAGCA. GAPDH-F: AGGTTGTCTCCTGTGACTTCAA, GAPDH-R: CTGTTGCTGTAGCCATATTCATTG. The level of gene expression was analyzed by the 2^−ΔΔCt^ method and normalized to that of actin.

#### Western blotting

The rat hypothalamus tissues were lysed in RIPA buffer with PMSF protease inhibitors (Amresco, USA) for 30 min on ice. The protein concentration of the lysates was measured using the BCA protein concentration determination kit (P0010, Beyotime, China). Equivalent amounts of protein were then separated electrophoretically on 10 to 15% SDS-PAGE gels and transferred onto PVDF membranes. After blocking with 5% milk in TBST for 1 h at room temperature, the membranes were incubated at 4 °C overnight with the primary antibodies (DRD2, ab85367; DRD3, ab42114; Adra1α, ab137123). The membranes were then washed three times using TBST and incubated for 1 h at room temperature with the secondary antibodies (goat anti-mouse IgG(H + L),1:2000, Beyotime, A0216; goat anti rabbit IgG (H + L), 1:1000, Beyotime, A0208). ECL buffer was added to the membranes; and the signals were visualized using a fluorescence imaging system (ChemiQ4600, Clinx Science,China).

#### Enzyme-linked immunosorbent assay

The ELISA kit (YT30086, YunTai Technology, China) was used to detect the level of DAT in the hypothalamus tissues according to the instructions of the manufacturer. Briefly, tissues were mixed with a ninefold volume of PBS and then grounded on ice, followed by centrifugation of the homogenate at 5000 g for 5 min. The supernatant was collected and tested using the ELISA kit.

### Statistical analysis

SPSS 20.0 and Graphpad prism 8.0 were used to perform the data analyses and graphic plotting. The data were expressed as mean ± standard deviation (SD). Student’s T test was used to compare differences between two groups, while analysis of variance (ANOVA) was used for the comparison of multiple groups. *P* < 0.05 was considered as a significant difference.

## Results

As shown in Table 1, the behavioristics of male rats were tested to analyze the function of ejaculation, sexual behavior, and sexual desire. Through the mating experiment, we found that the ejaculation latency time of the PE models (470.44 ± 64.88 s) was significantly less than that observed in the controls (764.2 ± 72.22 s, *P* =  < 0.05), while the latency time showed a significant increase after administration of sulpiride (758.63 ± 55.15 s), medium (737.56 ± 106.66) or high (744 ± 150.51 s) dose of Shugan Yidan fang (*P* < 0.05). The PE models (5 ± 1.23) also had more frequent ejaculations than the controls (2.9 ± 1.2, *P* < 0.05), with treatment with sulpiride (2.63 ± 1.41) or Shugan Yidan fang decreasing the frequency in a dose-dependent manner (low: 3.78 ± 0.83; medium: 3.11 ± 0.78; high: 2.38 ± 1.41, *P* < 0.05). In adtion, the intromission latency time in PE (21.33 ± 4.44 s) was significant shorter than that in the control group (31.8 ± 4.78 s, *P* < 0.05), while treatment with sulpiride (31.25 ± 6.92 s), medium (29.67 ± 6.93 s) and high (29.25 ± 6.43 s) dose of Shugan Yidan fang increased the time compared to the PE group (*P all* < 0.05). Similarly, the PE models administered the medium or high dose of Shugan Yidan fang recovered their intromission frequency (PE: 9.11 ± 1.9; medium: 15.44 ± 3.50; high: 17 ± 2.98, *P all* < 0.05). The mount latency and frequency were also recorded to evaluate sexual desire during the mating process. As shown in Table 1, the mount latency (8.11 ± 1.9 s) and frequency (8 ± 1.94) were both shorter or lower than those observed in the PE models (*P* < 0.05), with the medium (latency: 14.33 ± 4.06 s; frequency: 15.33 ± 4.06) or high (latency: 14.75 ± 3.45 s; frequency: 15.5 ± 2.88) dose of Shugan Yidan fang promoting recovery of the two parameters (*P* < 0.05). However, no statistically significant difference was observed between the PE group and the low-dose Shugan Yidan fang group. These results indicated that Shugan Yidan fang restored PE induced by piribedil in the rat models.

**Table 1:** Statistical analysis of different sexual behavior indexes of male rats in all matting groups

	Number	Ejaculation latency(s)	Ejaculation frequency (n)	Intromission latency(s)	Intromission frequency (n)	Mount latency (s)	Mount frequency (n)
Control	10	764.2 ± 72.22^#^	2.9 ± 1.2^#^	31.8 ± 4.78^#^	15 ± 1.7^#^	13.5 ± 3.06^#^	16 ± 2.67^#^
PE	9	470.44 ± 64.88^*^	5 ± 1.23^*^	21.33 ± 4.44^*^	9.11 ± 1.9^*^	8.11 ± 1.9^*^	8 ± 1.94^*^
PE + Sul	8	758.63 ± 55.15^#^	2.63 ± 1.41^#^	31.25 ± 6.92^#^	14.75 ± 2.82^#^	13.25 ± 4.97^#^	15.63 ± 2.83^#^
PE + low SY	9	482.44 ± 91.33^*^	3.78 ± 0.83^#*^	24.67 ± 5.59^*^	10.44 ± 3.84^*^	9.67 ± 3^*^	10.67 ± 4.42^*^
PE + medium SY	9	737.56 ± 106.66^#^	3.11 ± 0.78^#^	29.67 ± 6.93^#^	15.44 ± 3.50^#^	14.33 ± 4.06^#^	15.33 ± 4.06^#^
PE + high SY	8	744 ± 150.51^#^	2.38 ± 1.41^#^	29.25 ± 6.43^#^	17 ± 2.98^#^	14.75 ± 3.45^#^	15.5 ± 2.88^#^

Student’s T test was used to compare the statistical difference between the two groups

*PE* Premature ejaculation, *Sul* Sulpiride, *SY* Shugan Yidan fang

^#^significant difference compared to the model groups, *P* < 0.05

^*^significant difference compared to the control groups, *P* < 0.05

**Fig. 2:**
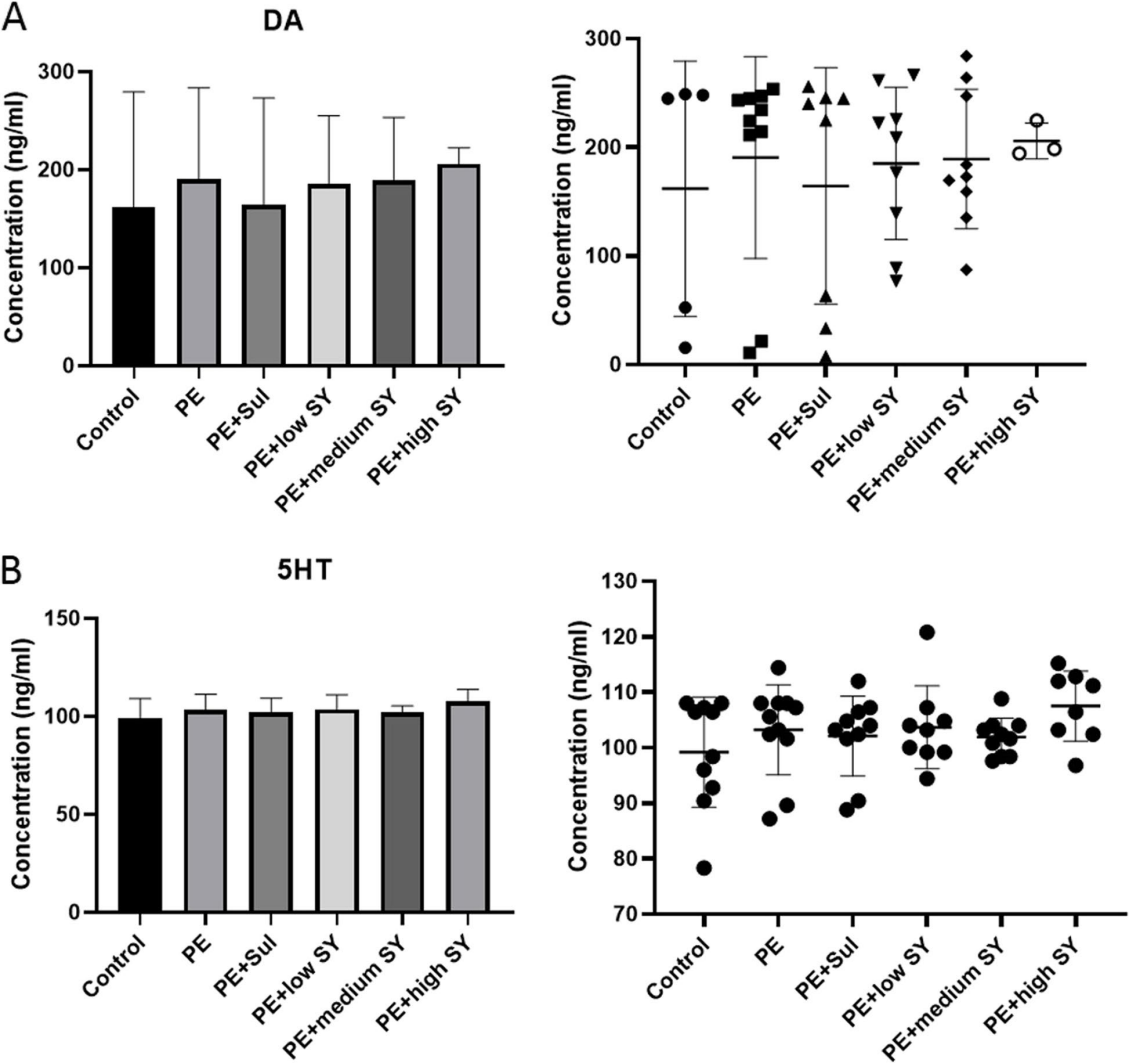
Dopamine and 5-HT levels were measured by reverse high-performance liquid chromatography. The histogram (left) and scatter diagram (right) show the DA (**A**) and 5-HT (**B**) levels in the hypothalamus of male rats in all studied groups. Student’s T test was used to compare the statistical difference between two groups. DA, Dopamine; 5-HT, 5-hydroxytryptamine; PE, premature ejaculation; Sul, sulpiride; SY, Shugan Yidan fang

**Fig. 3:**
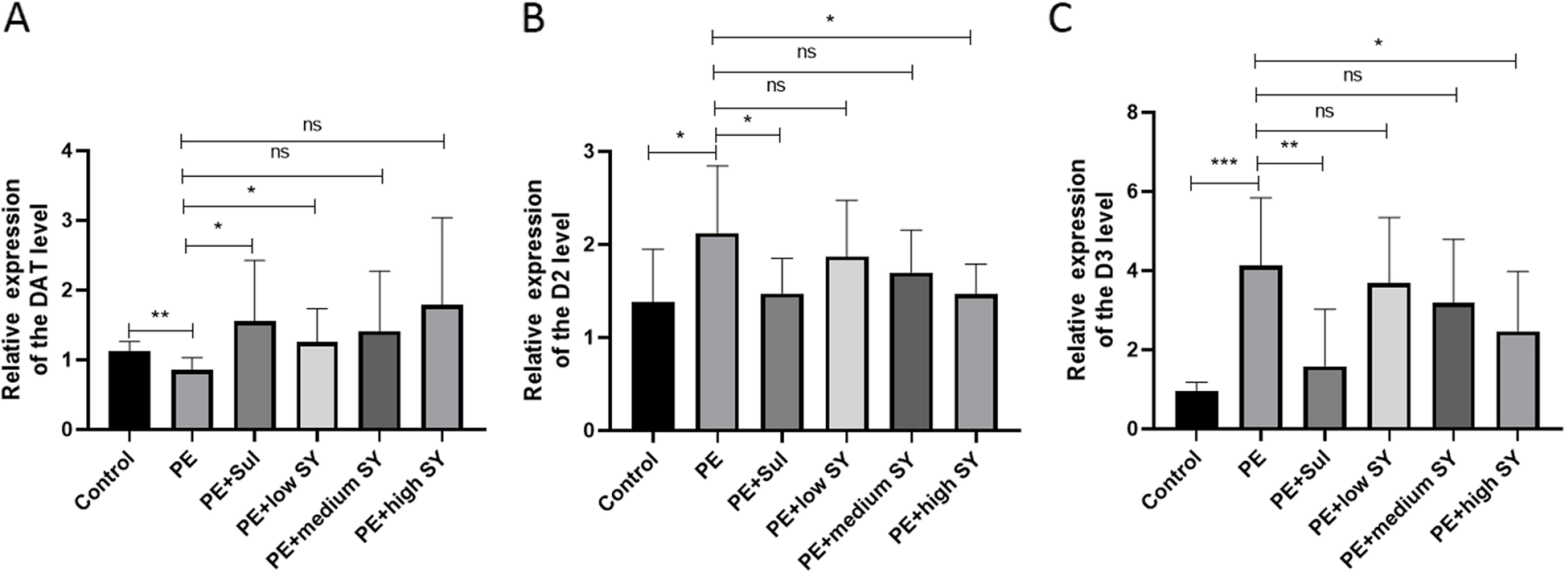
Histogram showing mRNA expression of the dopamine receptors and transporter. The mRNA expressions of DAT (**A**), D2 (**B**) and D3 (**C**) receptors of male rats were detected by RT-qPCR in all studied groups. Student’s T test was used to compare the statistical difference between two groups. *p < 0.05, **p < 0.01, ***p < 0.001, indicate a significant difference. Ns: no significant; DAT, dopamine transporter; D2/D3, dopamine D2/D3 receptors; PE, premature ejaculation; Sul, sulpiride; SY, Shugan Yidan fang

**Fig. 4:**
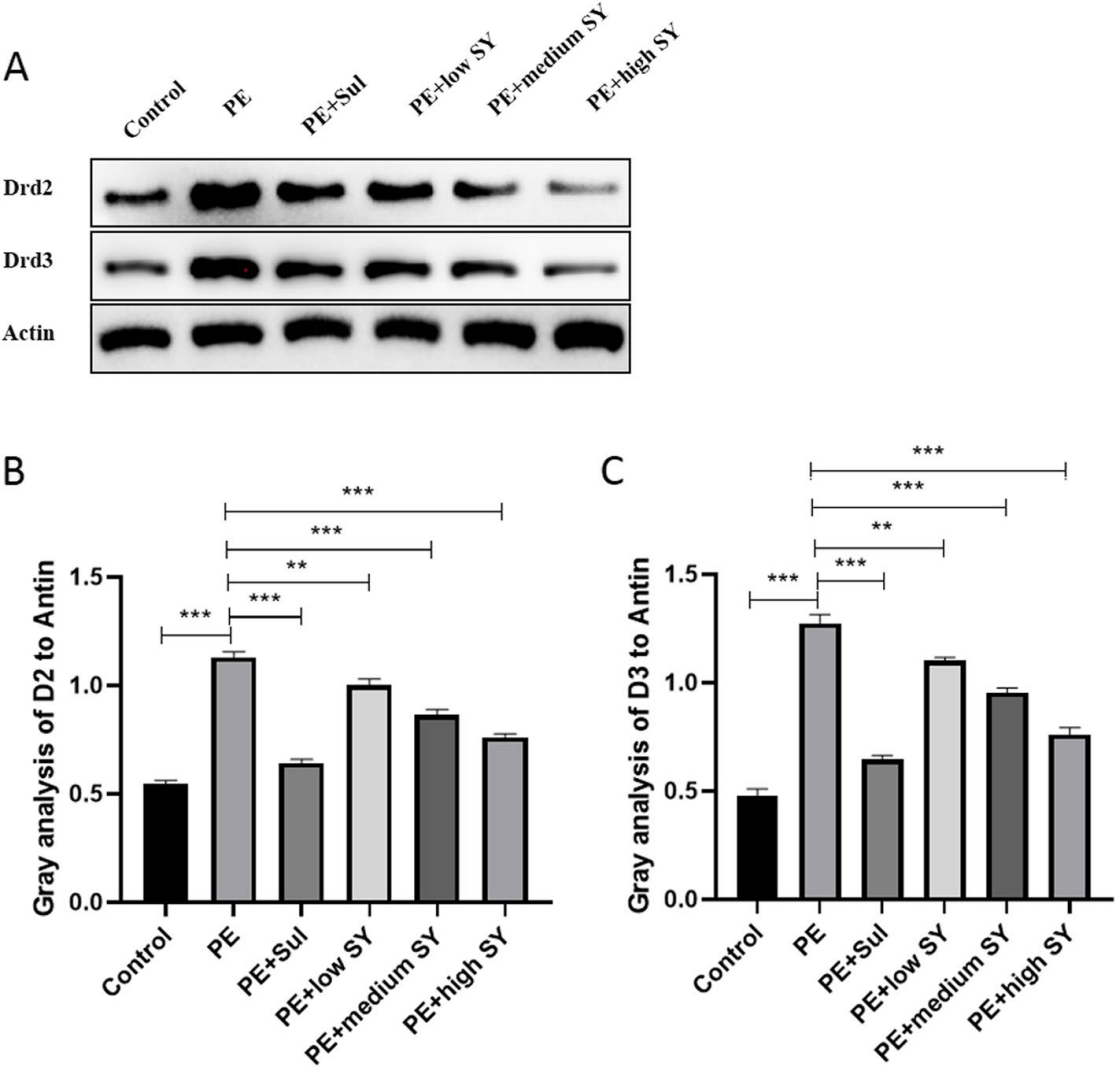
The protein level of the dopamine D2 and D3 receptors of male rats in all studied groups were detected by Western blots. **A** The electrophoretic band of D2/D3 and the reference gene, actin. **B** The value of gray analysis of D2/D3 to actin. Student’s T test was used to compare the statistical difference between two groups. **p < 0.01, ***p < 0.001, indicate a significant difference. Drd2/Drd3, dopamine D2/D3 receptors; PE, premature ejaculation; Sul, sulpiride; SY, Shugan Yidan fang

**Fig. 5:**
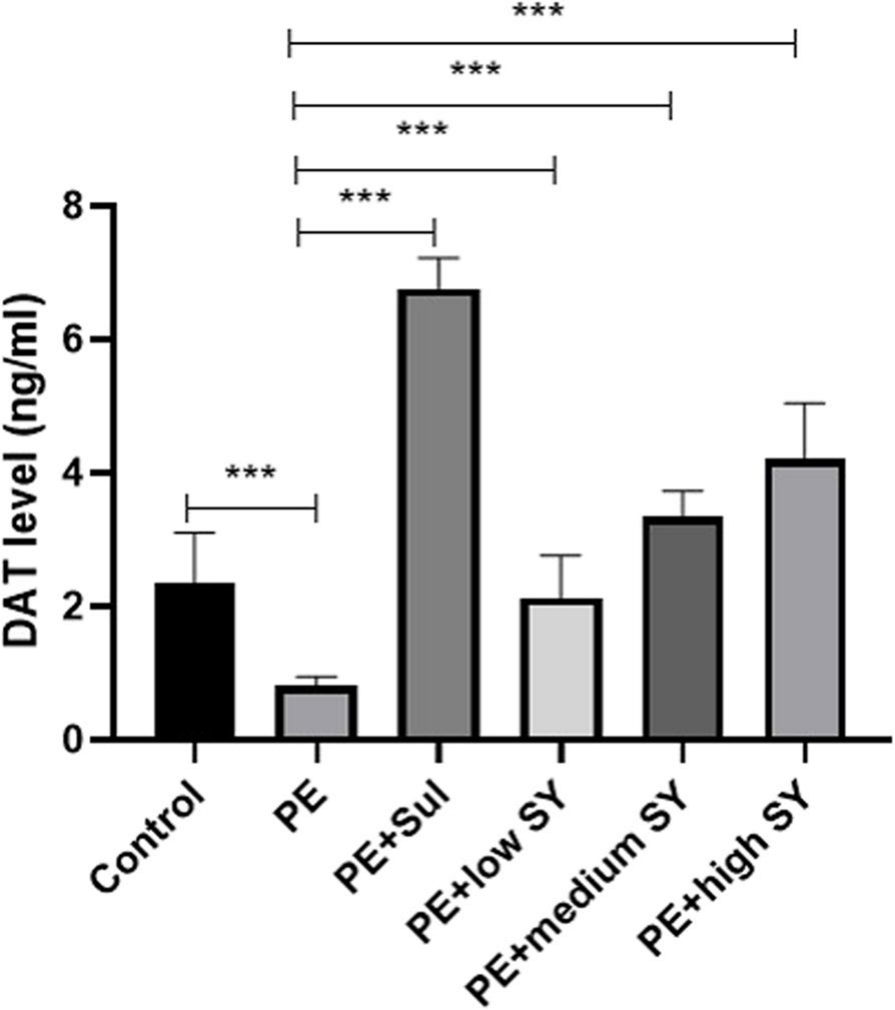
The DAT level in the hypothalamus of male rats in all the studied groups was detected by ELISA. Student’s T test was used to compare the statistical difference between two groups. ***p < 0.001, indicates a significant difference. DAT, dopamine transporter. PE, premature ejaculation; Sul, sulpiride; SY, Shugan Yidan fang

To further analyze the potential mechanism of Shugan Yidan fang on PE, the rats were sacrificed after the last mating test. We collected the hypothalamus tissues of each rat to determine the potential molecular mechanism. Firstly, the DA and 5-HT levels in the hypothalamus were measured by RP-HPLC. Although the DA level fluctuated, no significant difference was observed between the groups (Fig. 2). Similar to DA, no statistical difference was found in the level of 5-HT between the groups. However, the RT-qPCR results showed significantly low expression of DAT in the PE models than in controls (*P* < 0.05), while treatment with sulpiride or a low dose of Shugan Yidan fang significantly improved DAT expression (*P* < 0.05, Fig. 3A). The receptors of DA (D2/D3) showed higher expression in the PE model group than in the control group (*P* < 0.05), while adding sulpiride or a high dose of Shugan Yidan fang significantly reduced mRNA expression of the two receptors (*P* < 0.05, Fig. 3B and C). Moreover, the protein level of D2 and D3 were also detected by Western blotting. We showed that the protein levels of D2 and D3 increased significantly in the PE models compared to those in the control group (*P* < 0.05, Fig. 4), but decreased when treated with sulpiride or Shugan Yidan fang in a dose-dependent manner (*P* < 0.05, Fig. 4), similar to that observed for mRNA expression. The results of ELISA detection showed that the DAT level was decreased significantly in PE models compared to that in the controls (*P* < 0.05), Shugan Yidan fang resulted in a gradual recovery in DAT level in a dose-dependent manner (*P* < 0.05, Fig. 5). In addition, we also detected the alpha 1 adrenergic receptor for their important role in PE. The results showed that high level of Shugan Yidan fang reduced the mRNA expression of adra1α compared to PE groups, although no significant difference was found in protein level (Supplementary Fig. 1).

## Discussion

This study, confirmed that the Chinese herbal formula, Shugan Yidan fang restored ejaculation function, and improved the sexual function index in a rat model of PE. Our data showed that the mechanism of PE involved lower expression of DAT, with qPCR and ELISA technologies demonstrating that treatment with Shugan Yidan fang or sulpiride resulted in higher expression of DAT in the rats with PE. We also showed that expression of the DARs, D2/D3, was significantly higher in the PE group, while treatment with Shugan Yidan fang or sulpiride decreased the levels of these receptors as measured by qPCR and WB. Taken together, these results indicated that the potential mechanism of Shugan Yidan fang in PE therapy was associated with DA- DAT-DAR signal axis, that Shugan Yidan fang reduces DAR levels, thus reducing the excessive conduction of DA. Notably, the values of intromission frequency are greater than those of the corresponding mount frequency in our data. This is attribute to that the female mice had certain changes in their position after male mice inserted themselves, and although the male mice were still riding, the male penis slipped out and needed to be reinserted. 

In recent years, Chinese medicine formula has been shown to have potential for treating PE, however, there is limited research on the mechanism of its effect. Our study established an association between Shugan Yidan fang and the signals transmitted by DA. Studies have reported that DA is an essential neurochemical factor in the mediation of sexual functioning, as it promotes seminal emission and ejaculation via the D2 receptors [16]. Drugs targeting DA and its receptors have been investigated, with a clinical study demonstrating that DA-like compound extended ejaculation [17]. Although we didn’t find significant differences in DA among the different groups, GamalEl Din et al. [18] provided that fluoxetine administration significantly changed microRNAs 16 and 135a expression in the patients with premature ejaculation, indicating that these microRNAs are related to serotonin but not dopamine. Moreover, the contradiction to ours might also due to the different treatment drugs. Meantime, it has also been reported that DAR antagonists inhibit the intravaginal ejaculation latency time (IELT) induced by a DAR agonist [19]. Korotkova and his colleagues demonstrated that dopaminergic neuronal activity was inhibited by modafinil via the D2 receptors [20]. Similarly, Clément et al. reported that microinjection of a preferential D3 agonist, 7-OH-DPAT, into the medial preoptic area(MPOA)shortened IELT and promoted ejaculation in rats [21], This indicated that D3 may be a promising target for pharmacological treatment of PE. These earlier studies suggested that DA participates in the ejaculation disorders via the D2-like receptors, D2 and D3, conclusion consistent with the findings of our study.

Several studies have shown a relationship between PE and polymorphism of the DAT genes, such as SLC6A3 and DAT1 [22, 23]. A study by Eltonsi [24] reported that gene polymorphism of DAT determined the response of paroxetine and escitalopram to lifelong PE. These results indicated that heredity decrease in DAT may be a risk factor for the development of PE. As the key regulator of transmitter release and signaling dynamics, DAT can remove DA molecules from the extracellular and cytosolic space, both of which determine the amount of transmitter released from synaptic vesicles [25]. We demonstrated that Shugan Yidan fang restored the level of DAT in the PE rat models, which promoted transduction of DA to the functional regions. Notably, we found no significant difference in DAT mRNA levels between the PE models following administration of Shugan Yidan fang at medium or high doses. This result was probably attributable to the higher standard deviation of our data. Despite this possibility, our results provide an indicator reference for the use of Shugan Yidan fang. Also similar to GamalEl Din that showed miRNA 135a was associated with the response of fluoxetine [18], we consider that patients with a high level of DARs may benefit from administration of Shugan Yidan fang.

5-HT is associated with both sympathetic nervous system activity and sexual behavior [26]. Patients with PE have a higher serum level of 5-HT and studies have shown the important role of 5-HT receptors on modulation of ejaculation [27]. As described above, these neurotransmitters and receptors involved in the control of ejaculation are currently used as pharmacotherapy targets for patients with PE. Although there is clear evidence that DA and 5-HT participate in the control of ejaculation, we did not observe significant differences in the levels of DA and 5-HT between the PE model and treatment groups. We consider that this result may be due to the diverse temporal spatial distribution of these neurotransmitters in the development of PE. In fact, DA is synthesized mainly in the diverse encephalic region, including the hypothalamus, front of the midbrain, and spinal cord [28]. Neurons and their axons are involved in modulation of PE. For example, 5-HT, serotonergic neurons are distributed widely in the brain and spinal cord and are found predominantly in the brainstem, raphe nuclei, and the reticular formation [29]. We consider that the level of DA and 5-HT in the hypothalamus remains unchanged during the development of PE, and that other regions should be investigated to identify the main controller for the delivery of DA and 5-HT. We also propose a hypothesis that the level of DA is stabilized in the hypothalamus in the PE condition, and that transmission and amplification of these neurotransmitter signals are dependent on the downstream levels of DAR/DAT.

### Limitations of the study

Although we determined that the effect of Shugan Yidan fang on PE was associated with the DA transduction signal, we only focused on the level of DA in hypothalamus, and therefore other tissues such as different regions of the hypothalamus or cerebral cortex should be collected and analyzed. In addition, neurotransmitters or other inhibitors such as DAR agonists should be used to determine whether or not the therapeutic function of Shugan Yidan fang on PE can be reversed by these agents. Our study preliminarily provides evidence and a potential mechanism for the beneficial effect of Shugan Yidan fang on PE. Our findings therefore provide theoretical support for the clinical application of Shugan Yidan fang.

## Conclusions

In this paper, we report that the Chinese herbal medicine formula, Shugan Yidan fang, has beneficial effects in a rat model of PE by reducing the expression of DAR mRNA and protein expression whilst increasing the level of DATs in the hippocampus. These findings provide a molecular mechanism for the clinical application of Shugan Yidan fang in patients with PE.

### Supplementary Information

**Additional file 1: Supplementary Fig 1. **Detection of the expression of adrenergic receptor- adra1α by qPCR and western blots. A: The mRNA expression of alpha 1α were detected by qPCR between control, PE model and PE with high SY treatment of rats. B: The electrophoretic band of alpha 1α with the gray analysis were detected by western blots between control, PE model and PE with high SY treatment of rats. Student’s T test was used to compare the statistical difference between two groups. *p<0.05, **p<0.01, ***p<0.001, indicate a significant difference. ns: no significant. Adra1α: adrenergic, alpha-1A; PE, premature ejaculation; SY, Shugan Yidan fang.

## Data Availability

Materials have been listed in the methods section of article, the data that support the findings of this study are available from the corresponding author upon reasonable request.

## Abbreviations

DA: Dopamine
DAT: Dopamine transporter
DAR: Dopamine receptor
Adra1α: Adrenergic, alpha-1A
ELISA: Enzyme-linked immunosorbent assay. PE: premature ejaculation
qPCR: Duantitative real-time PCR
5-HT: 5-Hydroxytryptamine

## References

[1] Chung E, Gilbert B, Perera M (2015). Premature ejaculation: A clinical review for the general physician. Aust Fam Physician.

[2] Serefoglu EC, Saitz TR (2012). New insights on premature ejaculation: a review of definition, classification, prevalence and treatment. Asian J Androl.

[3] Gur S, Sikka SC (2015). The characterization, current medications, and promising therapeutics targets for premature ejaculation. Andrology.

[4] Veening JG, Coolen LM (2014). Neural mechanisms of sexual behavior in the male rat: emphasis on ejaculation-related circuits. Pharmacol Biochem Be.

[5] Giuliano F, Clément P (2006). Serotonin and premature ejaculation: from physiology to patient management. Eur Urol.

[6] Cinar O, Durmus N, Aslan G (2018). Effects of the dopamine D(3) receptor agonist 7-hydroxy-2-(di-N-propylamino) tetralin in hyperthyroidism-induced premature ejaculation rat model. Andrologia.

[7] Zhang JZ, Li HJ (2018). Progress in the treatment of premature ejaculation. Zhonghua Nan Ke Xue.

[8] Efimova EV, Gainetdinov RR, Budygin EA (2016). Dopamine transporter mutant animals: a translational perspective. J Neurogenet.

[9] Inden M, Takata K, Yanagisawa D (2016). α4 nicotinic acetylcholine receptor modulated by galantamine on nigrostriatal terminals regulates dopamine receptor-mediated rotational behavior. Neurochem Int.

[10] Deeh Defo PB, Asongu E, Wankeu MN (2017). Guibourtia tessmannii-induced fictive ejaculation in spinal male rat: involvement of D(1), D(2)-like receptors. Pharm Biol.

[11] Hussain SJ, Hameed A, Nazar HS (2010). Levosulpiride in premature ejaculation. J Ayub Med Coll Abbottabad.

[12] Liu Y, Yang LT, Long WJ (2020). Medication rules of traditional Chinese medicine for the treatment of premature ejaculation: an analysis based on data mining. Zhonghua Nan Ke Xue.

[13] Guo J, Wang F, Zhou Q (2021). Safety and efficacy of traditional Chinese medicine, Qiaoshao formula, combined with dapoxetine in the treatment of premature ejaculation: an open-label, real-life, retrospective multicentre study in Chinese men. Andrologia.

[14] Dan WU, Gao Y, Xiang H (2018). Exploration into mechanism of antidepressant of Bupleuri radix based on network pharmacology. Acta Pharm Sin B.

[15] Zhao ZY, Shen X, Ben-Xiang HU (2016). Systems pharmacology based researching of radix Bupleuri in treatment of different emotional diseases. Chin Pharm J.

[16] McMahon CG, Abdo C, Incrocci L (2004). Disorders of orgasm and ejaculation in men. J Sex Med.

[17] Heller J (1961). Another case of inhibition of ejaculation as a side effect of mellaril. Am J Psychiatry.

[18] GamalEl Din SAO, Motawi AAO, Rashed LA (2022). Study of the role of microRNAs 16 and 135a in patients with lifelong premature ejaculation receiving fluoxetine daily for 3 months: a prospective case control study. Andrologia..

[19] Jern P, Johansson A, Strohmaier J (2017). Preliminary Evidence for an Association Between Variants of the Catechol-O-Methyltransferase (COMT) Gene and Premature Ejaculation. J Sex Med.

[20] Korotkova TM, Klyuch BP, Ponomarenko AA (2007). Modafinil inhibits rat midbrain dopaminergic neurons through D2-like receptors. Neuropharmacology.

[21] Kitrey ND, Clément P, Bernabé J (2007). Microinjection of the preferential dopamine receptor D3 agonist 7-hydroxy-N, N-di-n-propylaminotetralin hydrobromide into the hypothalamic medial preoptic area induced ejaculation in anesthetized rats. Neuroscience.

[22] Safarinejad MR (2011). Relationship between premature ejaculation and genetic polymorphisms of the dopamine transporter gene (SLC6A3). BJU Int.

[23] Santtila P, Jern P, Westberg L (2010). The dopamine transporter gene (DAT1) polymorphism is associated with premature ejaculation. J Sex Med.

[24] Eltonsi TK, Tawfik TM, Rashed LA (2017). Study of the link between dopamine transporter gene polymorphisms and response to paroxetin and escitalopram in patients with lifelong premature ejaculation. Int J Impot Res.

[25] Mulvihill KG (2019). Presynaptic regulation of dopamine release: Role of the DAT and VMAT2 transporters. Neurochem Int.

[26] Gur S, Kadowitz PJ, Sikka SC (2016). Current therapies for premature ejaculation. Drug Discov Today.

[27] Gillman N, Gillman M (2019). Premature ejaculation: aetiology and treatment strategies. Med Sci.

[28] Koblinger K, Füzesi T, Ejdrygiewicz J (2014). Characterization of A11 neurons projecting to the spinal cord of mice. PLoS One.

[29] McMahon CG (2016). Emerging and investigational drugs for premature ejaculation. Transl Androl Urol.

