# Correction: Mechanism of Shugan Yidan fang, a Chinese herbal formula, in rat model of premature ejaculation

**DOI:** 10.1186/s12610-023-00216-9

**Published:** 2023-12-18

**Authors:** Qiang Han, Jun Guo, Renyuan Wang, Jiangminzi Li, Fu Wang, Qinghe Gao, Jiwei Zhang, Hetian Wang, Yin Zeng

**Affiliations:** 1 Department of Andrology, Dongcheng District, Beijing Hospital of Traditional Chinese Medicine, Capital Medical University, 23 Art Gallery Back Street, Beijing, China; 2 Department of Andrology, Xiyuan Hospital of China Academy of Traditional Chinese Medicine, Beijing, 100089 China; 3 Department of Endocrinology, Beijing Hospital of Traditional Chinese Medicine, Capital Medical University, Beijing, 100010 China

**Correction: Basic Clin Androl 33, 25 (2023)**

**https://doi.org/10.1186/s12610-023-00200-3**

Following publication of the original article [1], the authors reported an error in the title of the article. The original title “Mechanism of Shugan Yidan fan, a Chinese herbal formula, in rat model of premature ejaculation” should be replaced with “Mechanism of Shugan Yidan fang, a Chinese herbal formula, in rat model of premature ejaculation”. Principally, it should be Shugan Yidan fang, not Shugan Yidan fan.

We sincerely apologize for the error.

The original article [1] has been corrected.
