# Supplementary figures and images for: Mechanism of Shugan Yidan fang, a Chinese herbal formula, in rat model of premature ejaculation
Source: Basic Clin Androl. 2023 Oct 3;33:25. doi: 10.1186/s12610-023-00200-3 (PMC10546682; doi:10.1186/s12610-023-00200-3)

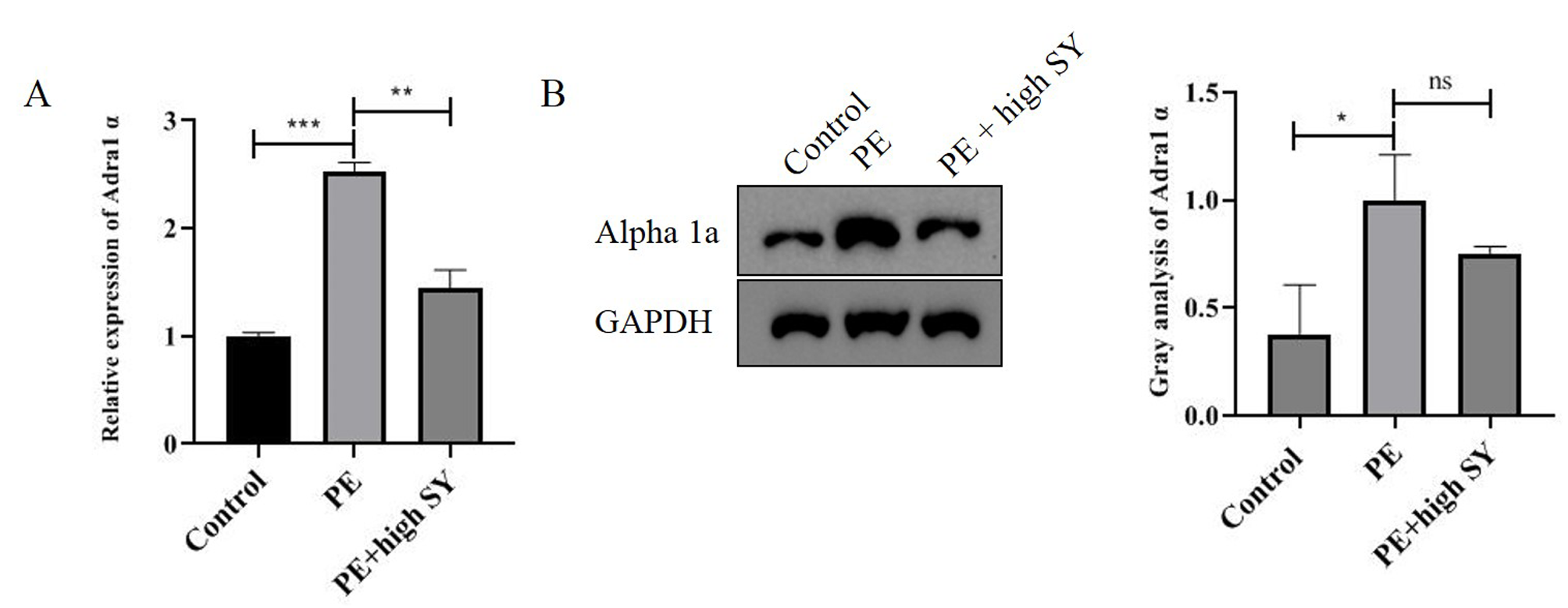

Supplement: Supplementary file 1 — Additional file 1: Supplementary Fig 1. Detection of the expression of adrenergic receptor- adra1α by qPCR and western blots. A: The mRNA expression of alpha 1α were detected by qPCR between control, PE model and PE with high SY treatment of rats. B: The electrophoretic band of alpha 1α with the gray analysis were detected by western blots between control, PE model and PE with high SY treatment of rats. Student’s T test was used to compare the statistical difference between two groups. *p<0.05, **p<0.01, ***p<0.001, indicate a significant difference. ns: no significant. Adra1α: adrenergic, alpha-1A; PE, premature ejaculation; SY, Shugan Yidan fang. [file 12610_2023_200_MOESM1_ESM.tif]
